# The causal relationship between gut microbiota and inflammatory dermatoses: a Mendelian randomization study

**DOI:** 10.3389/fimmu.2023.1231848

**Published:** 2023-09-27

**Authors:** Rui Mao, Qinyang Yu, Ji Li

**Affiliations:** ^1^ Department of Dermatology, Xiangya Hospital, Central South University, Changsha, Hunan, China; ^2^ Hunan Key Laboratory of Aging Biology, Xiangya Hospital, Central South University, Changsha, Hunan, China; ^3^ National Clinical Research Center for Geriatric Disorders, Xiangya Hospital, Central South University, Changsha, Hunan, China

**Keywords:** gut microbiota, two-sample mendelian randomization, psoriasis, rosacea, atopic dermatitis, vitiligo, eczema, acne

## Abstract

**Background:**

Observational studies have shown that gut microbiota is closely associated with inflammatory dermatoses such as psoriasis, rosacea, and atopic dermatitis (AD). However, the causal relationship between gut microbiota and inflammatory dermatosis remains unclear.

**Methods:**

Based on Maximum Likelihood (ML), MR-Egger regression, Inverse Variance Weighted (IVW), MR Pleiotropy RESidual Sum and Outlier (MR-PRESSO), Weighted Mode, and Weighted Median Estimator (WME) methods, we performed a bidirectional two-sample Mendelian randomization (MR) analysis to explore the causal relationship between gut microbiota and inflammatory dermatosis. The genome-wide association study (GWAS) summary data of gut microbiota came from the MiBioGen consortium, while the GWAS summary data of inflammatory dermatosis (including psoriasis, AD, rosacea, vitiligo, acne, and eczema) came from the FinnGen consortium and IEU Open GWAS project. Cochran’s IVW Q test tested the heterogeneity among instrumental variables (IVs). The horizontal pleiotropy was tested by MR-Egger regression intercept analysis and MR-PRESSO analysis.

**Results:**

Eventually, the results indicated that 5, 16, 17, 11, 15, and 12 gut microbiota had significant causal effects on psoriasis, rosacea, AD, vitiligo, acne, and eczema, respectively, including 42 protective and 34 risk causal relationships. Especially, Lactobacilli and Bifidobacteria at the Family and Genus Level, as common probiotics, were identified as protective factors for the corresponding inflammatory dermatoses. The results of reverse MR analysis suggested a bidirectional causal effect between AD and genus Eubacterium brachy group, vitiligo and genus Ruminococcaceae UCG004. The causal relationship between gut microbiota and psoriasis, rosacea, acne, and eczema is unidirectional. There was no significant heterogeneity among these IVs. In conclusion, this bidirectional two-sample MR study identified 76 causal relationships between the gut microbiome and six inflammatory dermatoses, which may be helpful for the clinical prevention and treatment of inflammatory dermatoses.

## Introduction

Inflammatory dermatoses represent a diverse group of diseases with multiple etiologies, including genetic factors, infections, and immune dysregulation (1), involving the activation of various immune cells and inflammatory mediators in both the innate and adaptive immune systems. Current studies have shown that gut microbiota may affect the host’s immune function. Under normal conditions, the interaction between gut microbiota and Toll-like receptors (TLR) on intestinal epithelial cells and immune cells facilitates homeostasis of the immune system (2). Gut microbiota may thus affect host skin immunity directly or indirectly through the gut-skin axis (3).

Various studies have shown significant differences in the gut microbial composition of patients with inflammatory dermatoses (4). For example, Bifidobacterium, considered a probiotic, decreases in eczema (5), acne (6), psoriases (7), and atopic dermatitis (AD) (8), but is enriched in rosacea (9). However, studies have varied in the relationship between gut microbiota and inflammatory dermatoses, such as Akkermansia muciniphila in psoriasis (10, 11). In observational studies, the association between the gut microbiota and inflammatory dermatoses is easily affected by confounding factors such as dietary patterns, environment, age, and lifestyle (12), making it challenging to draw a causal inference between gut microbiota and dermatoses.

The genome-wide association study (GWAS) establishes variant-trait associations by detecting genetic variation in individual genomes (13). Mendelian randomization (MR) integrates summary data from GWAS and explores causal relationships between exposure and outcomes by using exposure-related genetic variation as a substitute for exposure (14). Research design with MR follows the Mendelian inheritance law where “parental alleles are randomly assigned to offspring.” If genotype determines phenotype, the genotype is associated with a particular disease through the phenotype, and thus the association between inflammatory dermatoses and gut microbiota can be inferred using the genotype as an instrumental variable. MR is less prone to confounding factors because germline genetic variation is randomly assigned during meiosis and therefore reflects exposure without being affected by reverse causality. As an extension of the MR approach, bidirectional MR can be used to determine the direction of causality between two related phenotypes. In this study, we performed a bidirectional two-sample MR analysis of gut microbiota and six inflammatory dermatoses to reveal the causal relationships between gut microbiota and inflammatory dermatoses.

## Methods

### Data sources

This analysis employed the largest-scale gut microbiome genome-wide meta-analysis to date obtained from the international consortium MibioGen (15). The project studied the genome-wide genotypes and 16S ribosomal RNA gene sequencing of 18,340 participants in 24 cohorts from 11 countries, targeting variable regions V4, V3–V4, and V1–V2 of the 16S rRNA gene to delineate the microbial composition and to conduct classification using direct classification. Microbiome trait loci (mbTL) mapping was performed to identify genetic loci that affect the relative abundance in gut microflora. A total of 131 genera, 35 families, 20 orders, 16 classes, and 9 phyla with an average abundance of over 0.1% were included.

The GWAS summary data of Eczema came from the data released by the IEU Open GWAS project (GWAS ID: ieu-a-996, Trait name: Eczema) (16). The GWAS data included 11,059,641 single-nucleotide polymorphisms (SNPs) and 40,835 samples, including 10,788 in the case group and 30,047 in the control group. The GWAS summary data of Psoriasis (Number of SNPs = 16,380,464; ncase = 4,510; ncontrol = 212,242), Rosacea (Number of SNPs = 16,380,452; ncase = 1,195; ncontrol = 211,139), AD (Number of SNPs = 16,380,443; ncase = 7,021; ncontrol = 198,740), Vitiligo (Number of SNPs = 16,380,442; ncase = 131; ncontrol = 207,482), and Acne (Number of SNPs = 16,380,454; ncase = 1,299; ncontrol = 211,139) came from the data released by FinnGen consortium (GWAS ID: finn-b-L12_PSORIASIS, finn-b-L12_ROSACEA, finn-b-L12_ATOPIC, finn-b-L12_VITILIGO, and finn-b-L12_ACNE, Trait name: Psoriasis, Rosacea, Atopic dermatitis, Vitiligo, and Acne) (17). Age, sex, top 10 major components, and genotyping batches were corrected during the original author’s analysis (18). The details of the data sources used in this study are shown in **Table S1** (**Supplementary 1**).

### Instrumental variable (IV)

The bacterial taxa were classified into five hierarchical levels (phylum, class, order, family, and genus) to analyze, and each taxon was considered as a feature. SNPs associated with the gut microbiome were identified and used as instrumental variables. To ensure the authenticity and accuracy of the conclusions on the causal link between gut microbiome and inflammatory dermatoses risk, we selected instrumental variables (IVs) based on the following four criteria. First, the SNP-phenotype association level must reach the locus-wide significance threshold (P< 5 * 10^-8^) to select potential IVs. Unfortunately, only a small number of SNPs were selected as IVs. To explore more relationships between inflammatory dermatoses and gut microbiota to obtain more comprehensive results, the second threshold (P< 1 * 10^-5^) was used to identify SNPs which were selected as the second IV set. Second, SNPs with minor allele frequency (MAF) ≤ 0.01 were removed. Third, SNPs with R^2^ values<0.001 (clumping window size=10,000 kb) were filtered, and only the one with the lowest p-value was kept. Fourth, when palindromic SNPs existed, the allele frequency data were used to infer the forward-strand alleles.

### Statistical analysis

**Figure 1:**
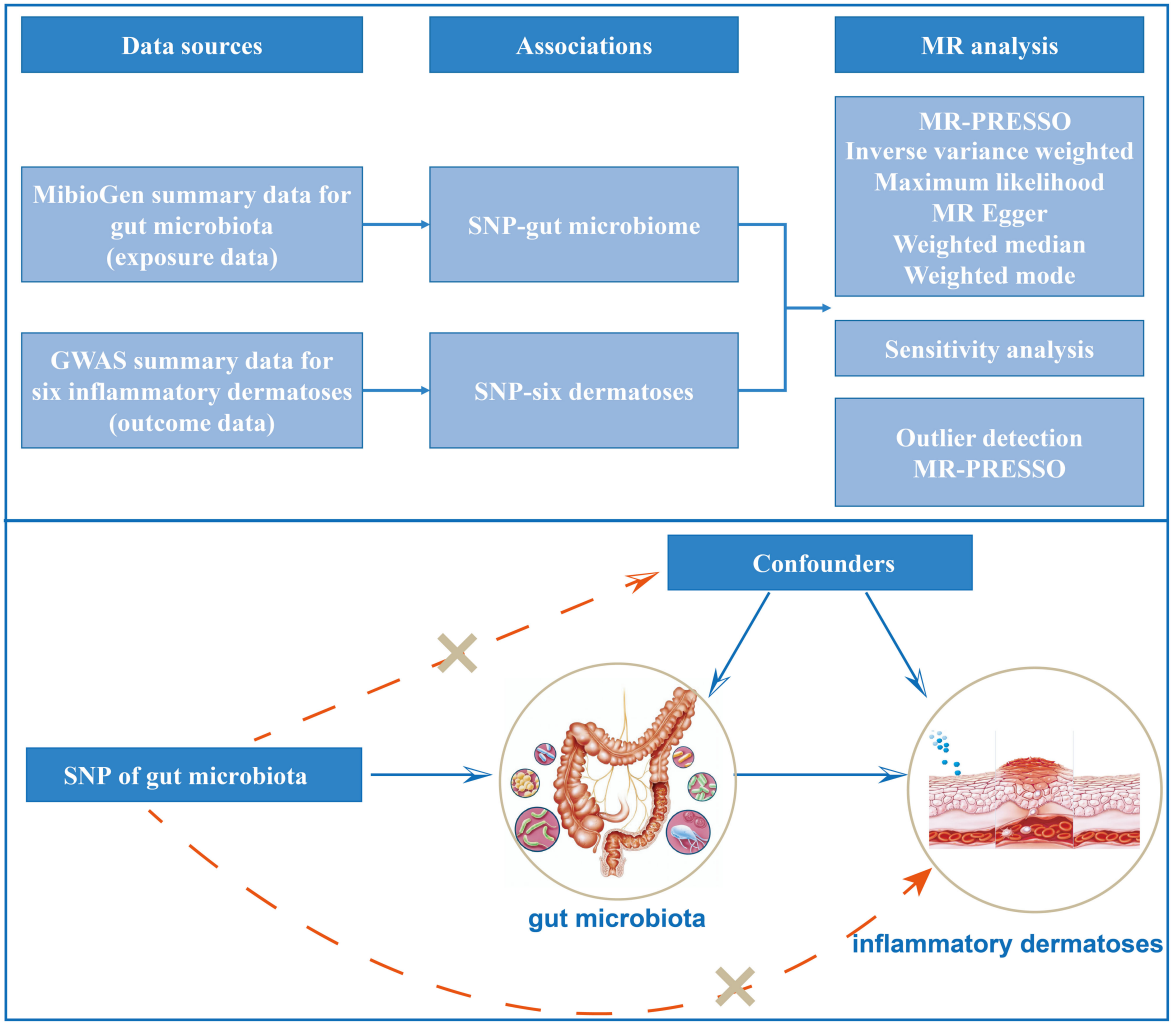
Study design of the two-sample Mendelian randomization for the effect of genetically predicted gut microbiome on inflammatory dermatoses.

The principle design of the whole study is shown in **Figure 1**. The detailed research flow chart and the three assumptions of Mendelian randomization analysis are presented in **Figure S1** of **Supplementary 2**. In this study, the following popular MR methods were used to examine whether there was a causal association between gut microbiota and inflammatory dermatoses: Inverse Variance Weighted (IVW) test, Maximum Likelihood (ML), Weighted Mode, MR-Egger regression, Weighted median estimator (WME), and MR Pleiotropy RESidual Sum and Outlier (MR-PRESSO). IVW test obtains the overall estimation of the influence of the gut microbiota on inflammatory dermatoses, by combining the Wald estimates for each SNP with a meta-analytic approach. It ignores the intercept term in regression and uses the inverse of outcome variance (se^2^) as the weight for fitting. If there is no horizontal heterogeneity, the IVW results would be unbiased (19). The ML method is analogous to IVW, and its standard error is smaller when the same assumptions are met (20). Based on the assumption of instrument strength independent of direct effect (InSIDE), MR-Egger regression is used to evaluate the existence of horizontal pleiotropy with intercept terms (21). If the intercept term is zero, it indicates no horizontal pleiotropy, and the MR-Egger regression result agrees with IVW (21). The WME method can estimate causality more correctly when more than 50% of the instrumental variables are invalid (22). When the InSIDE assumption is not fulfilled, the Weighted Mode has been shown to have a superior ability to detect a causal effect, with less bias and a lower type I error rate than MR-Egger regression (22). We also identified and corrected pronounced outliers with MR-PRESSO tests and MR-Egger regression (23).

Furthermore, we quantified the heterogeneity among the selected SNPs with Cochran’s *Q* statistic and identified potential heterogeneous SNPs with the “leave-one-out” analysis omitting each instrumental SNP in turn. Lastly, a reverse MR analysis of gut microbiota and inflammatory dermatoses was conducted with the same procedures and parameters as the forward MR.

In addition, we calculated the F statistic performed to assess the strength of the relevance between instrumental variables and exposure by the formula $F=\frac{R^{2}\times \left(n-k-1\right)}{\left(1-R^{2}\right)\times k}$ . R^2^ is the proportion of the variance of the trait accounted for by the SNP, k is the number of IVs, and n is the sample size (24). An F value over 10 indicates no significant weak instrumental bias. We utilized an online calculator tool available on https://shiny.cnsgenomics.com/mRnd/, developed by Marie-Jo A Brion et al., to determine the power of MR estimates (25, 26).

Furthermore, we utilized the PhenoScanner software to extract genes encompassing all identified SNPs and subsequently conducted a pathway enrichment analysis on those genes linked to SNPs contained within the instrumental variables of gut microbes demonstrating a significant causal association with inflammatory skin disorders (27). The enrichment analysis was executed using the clusterProfiler package (28). We omitted KEGG pathways with a P-value exceeding 0.05 and those with fewer than three mRNAs represented in the enrichment path.

False discovery rate (FDR) correction was conducted by applied fdrtools procedure, with a false discovery rate of FDR< 0.1 (29). All analysis was performed on R (version 4.2.2) and MR Analysis was based on MR-PRESSO (version 1.0) (23), TwoSampleMR (version 0.5.6) (30), and meta (version 6.2.1) packages (31).

## Results

### SNP selection

According to the selection criteria of IVs and removing the number of SNP repeated in different microorganisms, we identified 27 SNPs associated with gut microbiota at a significance level of p< 5 × 10^−8^. The details of 27 SNPs in the exposure and six outcome variables are shown in **Table S2** (**Supplementary 1**). Such few IVs is not enough for high-performance MR analysis. Therefore, under the screening of another threshold p< 1 × 10^−5^, we got 2,123 SNPs associated with gut microbiota in psoriasis, AD, rosacea, vitiligo, and acne, and 2,155 SNPs for eczema. The details of 2,155 SNP in the exposure and six outcome variables are shown in **Table S3** (**Supplementary 1**).

### Causal effects of gut microbiota on the development of six inflammatory dermatoses

In our study, we employed gut microbiota as an exposure and investigated its association with six inflammatory dermatoses using MR analysis. We filtered the IVs based on a threshold of p< 5 × 10^−8^. As indicated in **Table S4** (**Supplementary 1**), the limited number of available IVs restricted us to only two MR evaluation methods for analysis. However, the results showed that there was no causal relationship between the other five dermatoses and gut microbiota, except for acne, which had a causal relationship with a few gut microbiota. It is worth noting that MR analysis is not recommended when there are less than 3 IVs, as per the authoritative statement on MR analysis. Therefore, we opted to use IVs filtered by a threshold of p< 1 × 10^−5^ for subsequent MR analysis.

All identified gut microbiota with no less than 3 IVs were retained. The causal effects of the remaining 192 gut microbiota on psoriasis, rosacea, atopic dermatitis, vitiligo, and acne are shown in **Table S5** (**Supplementary 1**).

**Figure 2:**
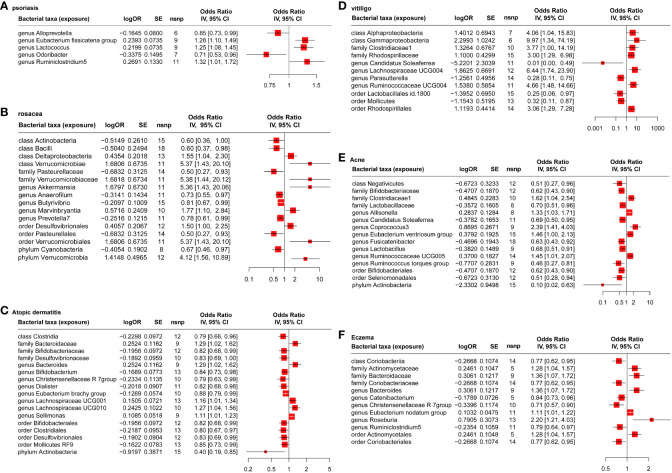
MR analysis of the causal relationship between genetically predicted gut microbiota and psoriasis **(A)**, rosacea **(B)**, atopic dermatitis **(C)**, vitiligo **(D)**, acne **(E)**, and eczema **(F)**.

Based on the estimate of IVW and ML, the result showed five, 18, 20, 14, 16, and 12 gut microbiotas had significant causal effects on psoriasis (**Figure 2A**), rosacea (**Figure 2B**), AD (**Figure 2C**), vitiligo (**Figure 2D**), acne (**Figure 2E**), and eczema (**Figure 2F**), respectively. It is worth noting that the common probiotics such as family Bifidobacteriaceae (OR = 0.82, 95%CI: 0.68–0.99, Pvalue = 0.0230, FDR = 0.0499) and genus Bifidobacterium (OR = 0.84, 95%CI: 0.73–0.98, Pvalue = 0.0197, FDR = 0.0289) have a protective effect on AD (**Figure 2C**). Order Lactobacillales (OR = 0.25, 95%CI: 0.06–0.97, Pvalue = 0.0447, FDR = 0.0757) had a protective effect on vitiligo (**Figure 2D**). In addition, family Bifidobacteriaceae (OR = 0.62, 95%CI: 0.43–0.90, Pvalue = 0.0118, FDR = 0.0319), family Lactobacillaceae (OR = 0.70, 95%CI: 0.51–0.96, Pvalue = 0.0159, FDR = 0.0260), and genus Lactobacillus (OR = 0.68, 95%CI: 0.51–0.91, Pvalue = 0.0068, FDR = 0.0103) have a protective effect on acne (**Figure 2E**).

**Figure S2** (**Supplementary 2**) presents a consolidated network highlighting gut microbiota implicated in several inflammatory skin conditions concurrently.

### Sensitivity analyses

As shown in **Table S5** (**Supplementary 1**), the explaining rate of the total variation (R^2^ values) of the 192 gut microbiota in six inflammatory dermatoses ranged from 0.40% to 10.58%, and the F values ranged from 12.27 to 139.83, excluding the possibility of weak genetic tool variables. Based on Cochran’s IVW Q test, there was no significant heterogeneity among these IVs (**Table S6** in **Supplementary 1**). In addition, according to the results of MR-Egger regression intercept analysis (**Table S7** in **Supplementary 1**), except for phylum Verrucomicrobia (p = 0.029) on rosacea, the other 205 intestinal microorganisms had no significant horizontal pleiotropy. However, further MR-PRESSO analysis (**Table S8** in **Supplementary 1**) did not find the horizontal pleiotropy in phylum Verrucomicrobia (Global test P-value = 0.356) on rosacea.

### Bidirectional causal effects between gut microbiota and six inflammatory dermatoses

Based on the selection criteria of IVs, we obtained 38, 14, 58, 7, 12, and 42 SNPs (P< 1 * 10^-5^, R^2^< 0.001) significantly associated with psoriasis, rosacea, AD, vitiligo, acne, and eczema, respectively. Summaries and details of each SNP are presented in **Table S9** (**Supplementary 1**). The results of reverse MR analysis (**Table S10** in **Supplementary 1**) showed that AD was causally associated with genus Eubacterium brachy group (OR = 1.41, 95%CI: 1.07–1.87, Pvalue = 0.0185, FDR = 0.0442, MR Egger). Vitiligo was causally associated with genus Ruminococcaceae UCG004 (OR = 0.97, 95%CI: 0.95–0.99, Pvalue = 0.0197, FDR = 0.0279, IVW), which indicated a bidirectional causal effect between them. No other significant causal relationship was found between six inflammatory dermatoses and the gut microbiota (**Table S10** in **Supplementary 1**). No notable heterogeneity was detected by Cochran’s Q statistics (**Table S11** in **Supplementary 1**, P > 0.05). The results of MR-Egger regression intercept analysis indicated a significant horizontal pleiotropy when we evaluated the cause-effect of AD on genus Eubacterium brachy group (p = 0.0217) (**Table S12** in **Supplementary 1**). But, further MR-PRESSO global test (**Table S13** in **Supplementary 1**) suggested no evidence of pleiotropy (Global test P = 0.566).

By investigating the genes aligned with the instrumental variables of gut microbes showing a marked causal association with inflammatory skin disorders (refer to **Table S14** in **Supplementary 1**) and conducting KEGG pathway enrichment analysis, we discerned a notable enrichment in inflammatory signaling pathways, including IL-17 signaling pathway, Chemokine signaling pathway and Cytokine-cytokine receptor interaction (refer to **Table S15** in **Supplementary 1**, **Figure S3** in **Supplementary 2**). This underlines the potential mechanisms by which gut microbes might influence the progression of inflammatory skin diseases via pathways like interleukins and cytokines.

## Discussion

This study constituted the first-ever attempt to explore a causal relationship between gut microbiota and inflammatory dermatoses using the summary statistics of the largest genome-wide meta-analysis of gut microbiotas conducted by the MiBioGen consortium. Based on the two-sample MR analysis, we identified 88 causal relationships between the gut microbiome and six inflammatory dermatoses.

Previous studies generally tended to consider increased gut microbiota in inflammatory dermatoses as potential risk factors and vice versa. Despite rich findings in the past (7), our MR analysis showed only five genera associated with psoriasis. Nam JH et al. reported that the genus Desulfovibrio decreased in patients with rosacea (32), while MR analysis showed that the order Desulfovibrionales was a risk factor. MR analysis indicated that among the gut microbiota of patients with AD, the protective factors contained the genus Bifidobacterium, family Desulfovibrionaceae, phylum Actinobacteria, etc. In addition, the findings about the genera Dialister and Bacteroides were consistent with previous studies (33–35). Limited studies of the gut microbiota in vitiligo have unveiled an association between disease duration and Ruminococcus, which is consistent with MR results (36). For acne, related studies found a decrease in the genera Bifidobacterium, Lactobacillus, Ruminococcaceae and phylum Actinobacteria (6, 37), which was consistent with our MR analysis results. For patients with eczema, MR analysis identified the genus Bacteroides and family Bacteroidaceae as risk factors while the genus Christensenellaceae R-7 group was a protective factor, which was consistent with the previous findings (5, 38, 39). In comparison, we can find that many findings are consistent, confirming the reliability of our MR results. However, the correlation between gut microbiota and inflammatory dermatoses can hardly be indicated based on the altered abundance of the gut microbiota.

The microbiota, identified as protective or risk factors, have also been much studied in their impact on inflammatory dermatoses. Bacteria such as Akkermansia, Ruminococcus, Bifidobacterium, Eubacterium, and Coprococcus are producers of short-chain fatty acids (SCFAs) (40, 41), including acetate, propionate, and butyrate. SCFAs are transported from the intestine to the skin via peripheral circulation. They modulate the function of immune cell function to reduce inflammatory factor release (42), improve mitochondrial function (43), and promote keratinocyte metabolism and differentiation (43). These effects are achieved through binding to G protein-coupled receptors (GPCR) and peroxisome proliferator-activated receptor gamma (PPARγ) (42, 44), promoting mitochondrial fatty acid β-oxidation (FAO) (45), and inhibiting histone deacetylase (HDAC) (46). Epidermal keratinocytes can metabolize butyrate into long-chain fatty acids (LCFAs) and very long-chain fatty acids (VLCFAs), which contribute to ceramide synthesis for skin barrier repair (43). In addition, SCFAs benefit the intestinal barrier (47). Topical and oral administration of SCFAs or their derivatives have also been found to be effective in treating inflammatory dermatoses (48). Lactobacillus and Bifidobacterium may inhibit immune inflammation by increasing tryptophan (Trp) and Trp metabolites, maintaining intestinal barrier function on the one hand and reducing acne inflammation on the other (49, 50). Bifidobacterium longum can metabolize Trp to indole-3-carbaldehyde (I3C), activating the aryl hydrocarbon receptor (AHR)-mediated immune signaling pathway, suppressing Th2 cells, and thus relieving AD (51). Sulfate-reducing bacteria (SRB), including Desulfovibrionaceae, convert sulfate in the intestine to hydrogen sulfide (H_2_S), which impairs epithelial barrier function by interfering oxidation of butyrate in the colon, with subsequent disruption of intestinal permeability (52). Bifidobacterium, Lactobacillus, and Roseburia spp metabolize polyunsaturated fatty acids, including omega-3 and omega-6 fatty acids, to conjugated linoleic acid (CLA) (53, 54), thereby inhibiting COX-2/5-LOX and TLR4/NF-κB signaling pathway and attenuating skin lesions of AD (55). Oral administration of Lactobacillus can reduce insulin-like growth factor 1 (IGF-1) and increase forkhead box protein O1 (FoxO1) expression in the skin, improving the condition of acne (56). However, the microbiota of similar lineages may bring different outcomes for the same or different inflammatory dermatoses. For example, Ruminococcus produces SCFAs which can alleviate inflammatory dermatoses. However, the genera Ruminococcaceae UCG004 and Ruminocaccaceae UCG005 were identified as risk factors for vitiligo and acne, respectively, while the genus Ruminococcus torques group was identified as a protective factor for acne. Future studies should clarify the effects of different microbiota for different dermatoses.

Existing findings have pointed out a potential avenue for treating inflammatory dermatoses by manipulating the gut microbiota. From a dietary standpoint, dietary composition and bioactive substances can influence inflammatory dermatoses by altering the gut microbial structure (57–59). The intake of probiotics, prebiotics, and synbiotics also alleviates inflammatory dermatoses (60). It is important to emphasize that the common probiotics, Lactobacillus and Bifidobacterium were identified as protective factors for the corresponding inflammatory dermatoses. After receiving fecal microbiota transplantation (FMT) from healthy mice, AD mice experienced changes in their gut microbiota, including a significant increase in the family Desulfovibrionaceae and the genus Lactobacillus. The level of SCFA in the feces of AD mice increased after FMT, balancing the abnormal immune responses and alleviating AD skin lesions (61).

The present study has the following strengths. First, our study is the first to assess the bidirectional causal relationship between gut microbiota and inflammatory dermatoses using a two-sample MR analysis. Second, the results of the two-sample MR analysis were less susceptible to confounding factors, reverse causality, and exposure than in the observational study. Third, the strength of instruments in the MR analysis was ensured by using the most extensive available GWAS meta-analysis of the MiBioGen consortium; sensitivity analysis was performed to ensure the consistency of causal estimation and the robustness of the results; the detection and exclusion of horizontal pleiotropy by using MR-PRESSO and MR-Egger regression intercept term tests.

However, there are some limitations to this study. First, the analysis used pooled data of disease types and therefore did not allow for subgroup analysis around disease subtype and severity. Second, the main participants in GWAS are from Europe, so the extrapolation of results to other ethnicities will be limited. Third, the SNPs used in the analysis did not reach the traditional GWAS significance threshold (P< 5 × 10^-8^). Still, we needed to include more genetic variation as IV for sensitivity analysis and horizontal pleiotropy detection. When the P value is set to 5 × 10^-8^ or 1 × 10^-6^, only one or no SNP is available for each microbe. For this, we used FDR correction to restrict the possibility of false positives. There are a lot of high-level studies screened in this way (15, 62). Finally, the bacterial groups were analyzed only at the order or family level. Future studies using more advanced metagenome sequencing analysis will produce more specific and accurate results.

## Conclusion

In conclusion, the results of our two-sample MR analysis support a potential causal relationship between gut microbiota and six inflammatory dermatoses. Various probiotics, including Lactobacillus and Bifidobacterium, have been shown to have protective causality against inflammatory dermatoses. However, the gut microbiota of similar lineages or that share the same characteristics in some way may bring different outcomes for the same or different inflammatory dermatoses. The results of this study deepen the understanding of the “gut-skin axis” and help to prevent and treat inflammatory dermatoses by regulating the structure of the gut microbiota.

## Data availability statement

The datasets presented in this study can be found in online repositories. The names of the repository/repositories and accession number(s) can be found in the article/**Supplementary Material**.

## Author contributions

RM: Conceptualization, Methodology, Software, Investigation, Visualization, Writing an original draft. QY: Methodology, Writing an original draft. JL: Conceptualization, Writing - original draft, Funding acquisition, Supervision. All authors contributed to the article and approved the submitted version.
