# Supplementary material for: The causal relationship between gut microbiota and inflammatory dermatoses: a Mendelian randomization study
Source: Front Immunol. 2023 Sep 27;14:1231848. doi: 10.3389/fimmu.2023.1231848 (PMC10565349; doi:10.3389/fimmu.2023.1231848)
Supplement: Supplementary file 2 [file DataSheet_1.docx]

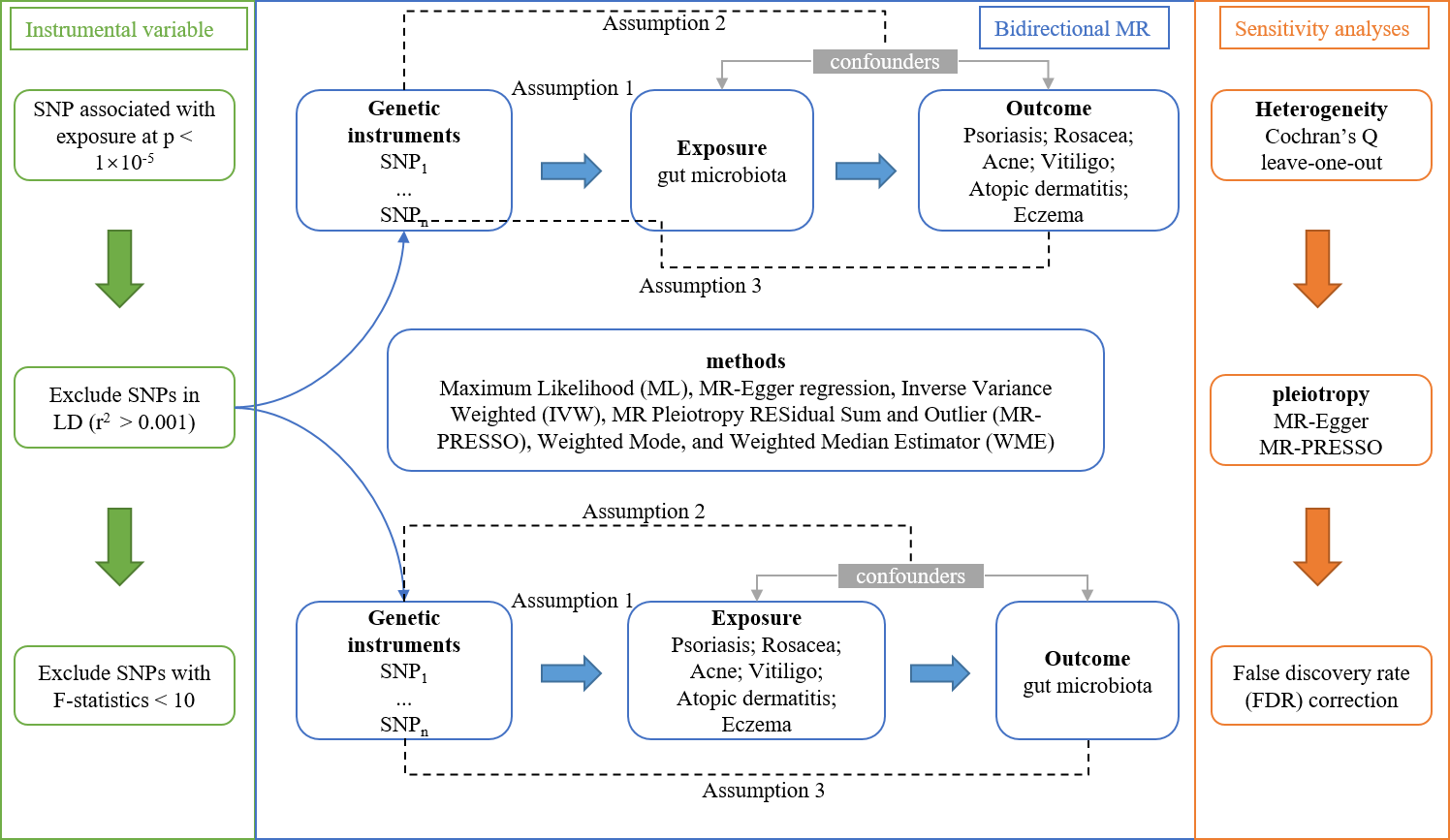


**Figure S1**. Study design overview and assumptions of the Mendelian randomization (MR) design. Dashed lines represent potential pleiotropic or direct causal effects between variables that would violate MR assumptions. Assumption 1: genetic variants are associated with the exposure; Assumption 2: genetic variants are not associated with any confounders; and Assumption 3: genetic variants influence risk only through the exposure and not through any alternative pathways.


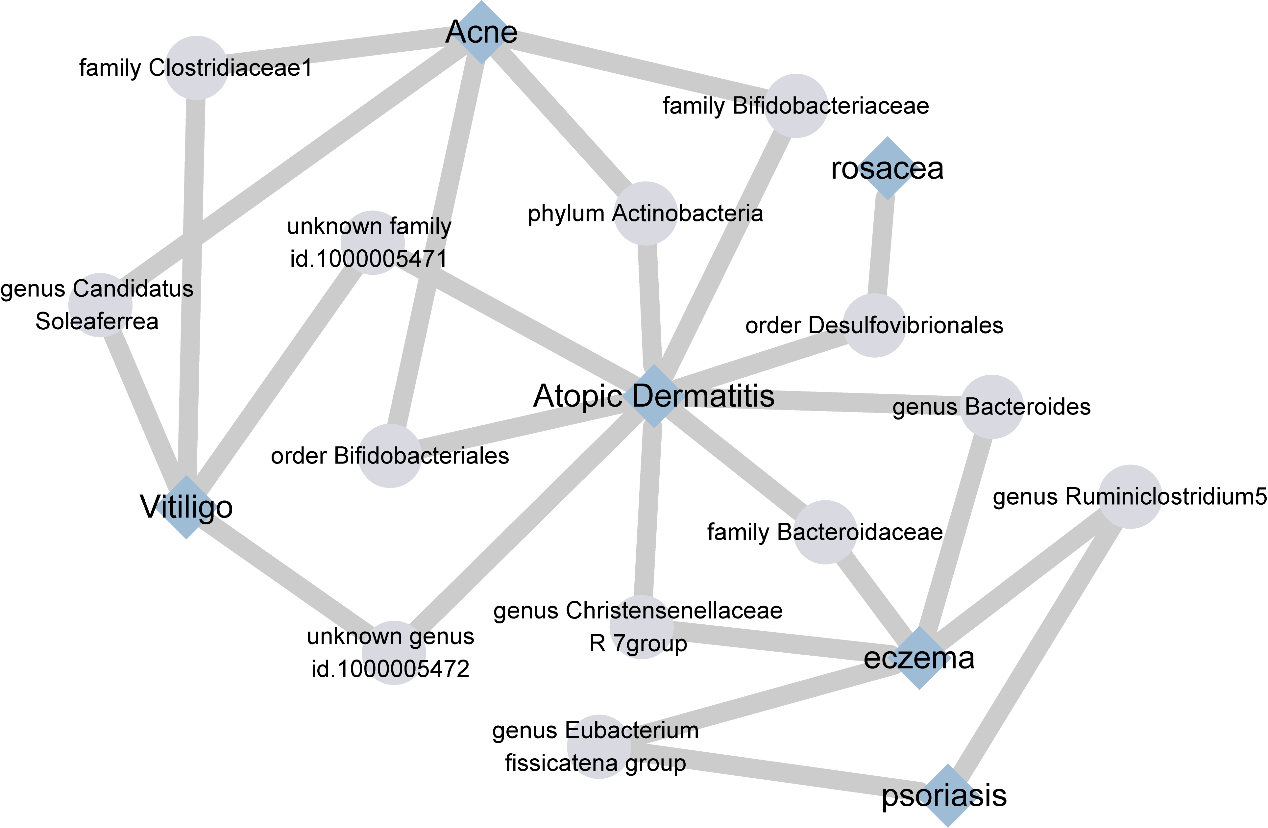


**Figure S2.** Gut microbiota that have a significant causal relationship with two or more inflammatory dermatoses.


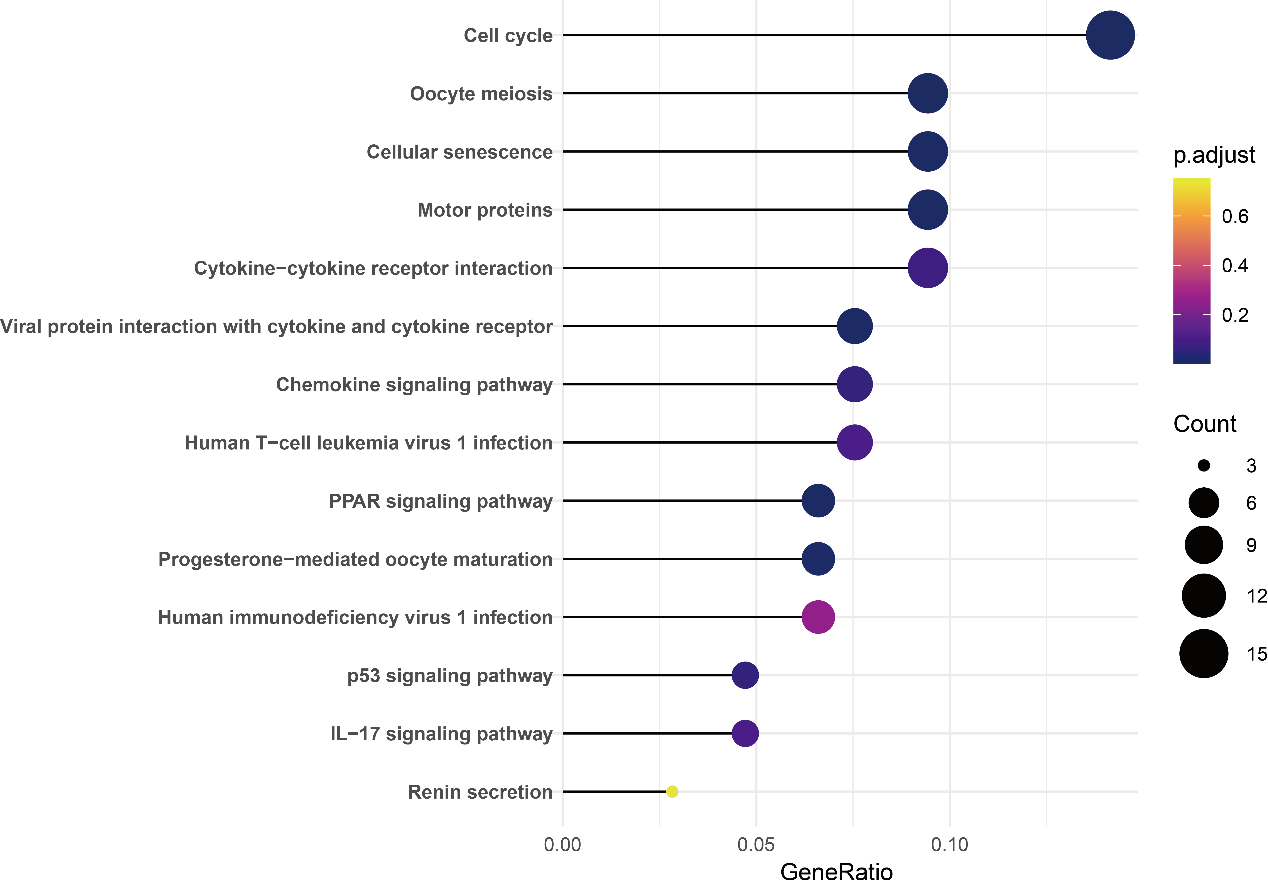


**Figure S3.** KEGG enrichment analysis of genes where SNPs included in instrumental variables are located.
